# Continuous preparation of antimony nanocrystals with near infrared photothermal property by pulsed laser ablation in liquids

**DOI:** 10.1038/s41598-020-72212-2

**Published:** 2020-09-15

**Authors:** Juanrong Kou, Yongkai Wang, Xiaoyu Liu, Xianju Zhang, Gaoyu Chen, Xiangxing Xu, Jianchun Bao, Kaili Yang, Lihui Yuwen

**Affiliations:** 1 School of Chemistry and Materials Science, Nanjing Normal University, Nanjing, 210046 China; 2 Key Laboratory for Organic Electronics and Information Displays and Jiangsu Key Laboratory for Biosensors, Institute of Advanced Materials (IAM), Jiangsu National Synergetic Innovation Center for Advanced Materials (SICAM), Nanjing University of Posts and Telecommunications, Nanjing, 210023 China

**Keywords:** Nanoparticles, Synthesis and processing

## Abstract

Antimony nanocrystals (Sb NCs) are of interest in energy storage, catalysis and cancer therapy for its special physical, chemical and biomedical properties. However, methodology challenges still remain in preparation of colloidal Sb NCs, due to the restricted reaction solution systems, high temperature and time costing for common routes. Herein, size controllable colloidal Sb NCs were continuously prepared by pulsed laser ablation of Sb target in different solvents, owning to the metal nanodroplet explosive ejection and thermal evaporation mechanisms. These well dispersed and stable Sb NCs showed excellent photothermal property in the near-infrared-II window.

## Introduction

When the external-, internal- or surface-structure of a material in at least one dimension is featured in the scale range approximately from 1 to 100 nm, it can be called nanomaterial. Compared with their bulk corresponding materials, the nanomaterials may have unique chemical, physical and biological properties due to their highly tailorable small size, morphology, component, structure and surface^1–6^. The state of the art of the nanomaterials has been connected with almost all scientific fields and industries, from energy to agriculture, from information to life science, and from basic physics to cosmic exploration. Antimony is a shiny, hard and brittle metal with a layered crystal structure. In each layer, it contains wrinkled six-member rings forming distorted honeycomb lattice structure^7^. The decrease of the Sb size to nanometer scale may bring out tailorable properties, e.g. the enhanced rate-capability and higher cycling stability as anode materials in rechargeable batteries^8–17^, the higher photothermal conversion efficacy achieved in near infrared photothermal cancer therapy^18–22^, the increased catalytic properties^23^, and the tuned band structure with the reduction of crystal plane layers from few to monolayer^24–26^. Various methods have been developed to prepare Sb nanomaterials, for examples, the room temperature reduction or high temperature solvothermal synthesis routes toward colloidal Sb NCs^27,28^, the solid sublimation or reduction methods to prepare Sb NC nano composites^29–32^, the intercalation chemical, electrochemical, sonochemical and mechanical exfoliation strategies to prepare antimonene^33–40^. However, challenges and limitations still remain in preparation methods of colloidal Sb NCs. (1) To synthesize size controllable Sb NCs, it needs careful selection of the reaction solution system with proper viscosity, polarity and volatility, deliberated choice or trying of various precursors, surfactants, reaction temperature and time. The inert atmosphere protection is also need especially for reaction under elevated temperature. (2) After the synthesis, repeated washing to eliminate the by-products is required, during which the small nanosized Sb may not easily separated, and the washing may also lead to nanocrystal aggregation. Post-synthesis transfer of the colloidal Sb NCs to different solvents may needs additional surface modification. (3) Continuous preparation requirement. The last but not the least, the batch by batch synthesis is less applicable for mass production due to the time costing and difficulty in quality control when scaling up. A summary on typical preparation of Sb nano materials is shown in Table 1.

**Table 1:** Typical preparation methods of Sb nano materials.

Sb morphology	Size	Preparation methods	References
Nanocrystals	~ 5–50 nm tunable	Pulsed laser ablation in liquids (PLAL)	This work
Nanocrystals	~ 10 − 20 nm	Solvothermal	^28^
Nanocrystals	~ 20 nm	Solvothermal	^27^
Nanocrystals	~ 34 nm	NaBH_4_ reduction	^21^
Nanocrystals	4 nm	NaBH_4_ reduction	^41^
Nanocrystals in carbon microsphere	< 10 nm (C sphere ~ 0.5 μm)	Spray pyrolysis	^11^
Nanocrystals in carbon nanosheets	~ 100 nm	Sol–gel + thermal treatment	^12^
Bundle-like nanorods	(50–80) * (200–300) nm	Solvothermal	^18^
Rod-like nanocrystals (in carbon fibers)	Diameter 10–30 nm	Electrospinning + high temperature reduction	^8^
Nanocrystals (in carbon fibers)	4 nm	Electrospinning + high temperature reduction	^9^
Antimonene nanosheets	0.5–1.5 μm (thickness 5–30 nm)	Solution-phase synthesis	^33^
Sb/antimonene nanoparticles	~ 55 nm	Liquid exfoliation (Ultrasound probe sonication)	^22^
Antimonene dots	~ 2.8 nm (Thickness: ~ 1.6 nm)	Liquid exfoliation (ultrasound probe sonication)	^19^
Antimonene dots	2.4 ± 1.2 nm (Thickness: 1.6–2.5 nm)	Liquid exfoliation (ultrasound probe sonication)	^20^
Antimonene dots	3.4 nm (Thickness: 3.2 nm)	Liquid exfoliation (ultrasound probe sonication)	^42^
Antimonene nanosheets	~ 108.0 nm (Thickness: ~ 4.5 nm)	Liquid exfoliation (ultrasound probe sonication)	^20^
Sb nanosheets	~ 350 nm (Thickness: ~ 3.5 nm)	Cathodic exfoliation	^40^

In this report, pulsed laser ablation in liquid medium (PLAL) method was applied to prepare Sb NCs matching all these challenges. The unique advantages of this preparation technique include: (1) the Sb NCs can be prepared by PLAL on Sb target in various solvents. Surfactants or ligands can be introduced, but it is not a necessary condition. Well dispersed Sb NCs can be achieved in solvents without additional surfactants. (2) The Sb NCs can be fast and continuously prepared, which is easy for scale up production, with the uniform quality of the product. The wavelength of the pulsed laser was 1,064 nm with maximum output power 30 W. Two mechanisms of the nanodroplet explosive ejection and thermal evaporation were demonstrated for the Sb NC formation with PLAL. The former yields Sb NCs of tens of nanometers, while the later produces smaller Sb NCs. The domination of either mechanism was successfully controlled by the laser power and solvents. The near infrared photothermal property of the Sb NCs were investigated.

## Experimental

### Materials

Oleylamine (80–90.0%) and *N*-Methyl-2-pyrrolidone (NMP, AR, 99.0%) were purchased from Aladdin. Oleic acid (90.0%) was purchased from Alfa Aesar. Sodium borohydride (99.0%) was purchased from Fluka. All other chemical reagents including antimony (5N), dodecanethiol (CP), ethanol (AR), methanol (AR), n-butanol (AR), ethylene glycol (AR), isopropanol (AR), glycerol (AR), cyclohexane (AR), acetone (AR), polyvinylpyrrolidone (PVP, K-30), *N*,*N*-dimethylformamide (DMF, AR) and antimony trichloride (AR) were purchased from Sinopharm Chemical Reagent Co. Ltd. All the reagents were used without further purification.

### Instruments

The Sb plate was prepared by a mechanical tablet press machine (JX-1). The PLAL was carried out by a pulsed laser with wavelength 1,064 nm and maximum output power 30 W (JW-F30). The continuous preparation was carried out by using the smart syringe pumps (XMSP-2A, Nanjing XiMai nanotech. Co. Ltd.) The morphology of the Sb NCs was measured by transmission electron microscopy (TEM, HITACHI H-7650) with the accelerating voltage 80 kV. The high resolution TEM (HRTEM) images were taken on JEOL-2100F with the accelerating voltage of 200 kV. The X-ray diffraction (XRD) patterns were recorded using a D/max 2,500 VL/PC diffractometer equipped with graphite monochromatized Cu Kα radiation (λ = 0.15406 nm). The absorption spectra were measured by a PerkinElmer spectrometer Lambda 950. The 1,064 nm laser (MIL-N-1064) with the power density of 1 W/cm^2^ was used for the photothermal measurement. The temperature change was recorded by using an infrared thermal camera (Fortic225, IRS Systems Inc.).

### Continuous preparation of Sb NCs

**Figure 1:**
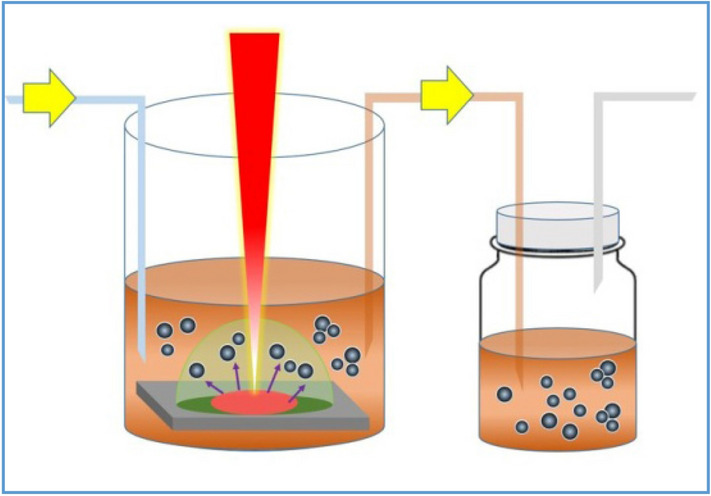
Schematic illustration of the PLAL preparation of colloidal Sb NCs.

The Sb NCs were prepared by the PLAL method. First, proper amount of Sb powder was compressed into a plate by a mechanical tablet press machine. Then the Sb plate was used as the ablation target of a pulsed laser with wavelength of 1,064 nm and output power of 30 W if not specifically mentioned. The pulse width and frequency of the laser were fixed as 1 μs, 5 kHz and the focus spot set to be 20 μm. Typically the target Sb plate was placed at the bottom of a 50 mL beaker, completely submersed in a solvent of 10 mL. The laser focus was adjusted to the plate surface. The ablation area was set to be a 5 mm × 5 mm square. During the laser ablation, the colorless solution was changed to brown in couple of seconds, indicating the formation of Sb NCs. The colloidal Sb NCs solution was pumped to the collection container; simultaneously the pure solvent was pumped in with the same flow rate, revealing the continuous preparation. The schematic illustration of the preparation is shown in Fig. 1. During the preparation, the laser light path shall not be blocked before the target due to the high energy density of the laser and shall be carried out in a ventilation condition. The Sb NCs can be precipitated by centrifugation for further characterization. The size of Sb NCs was controlled by adjusting lasing power and the solvent.

## Results and discussions

**Figure 2:**
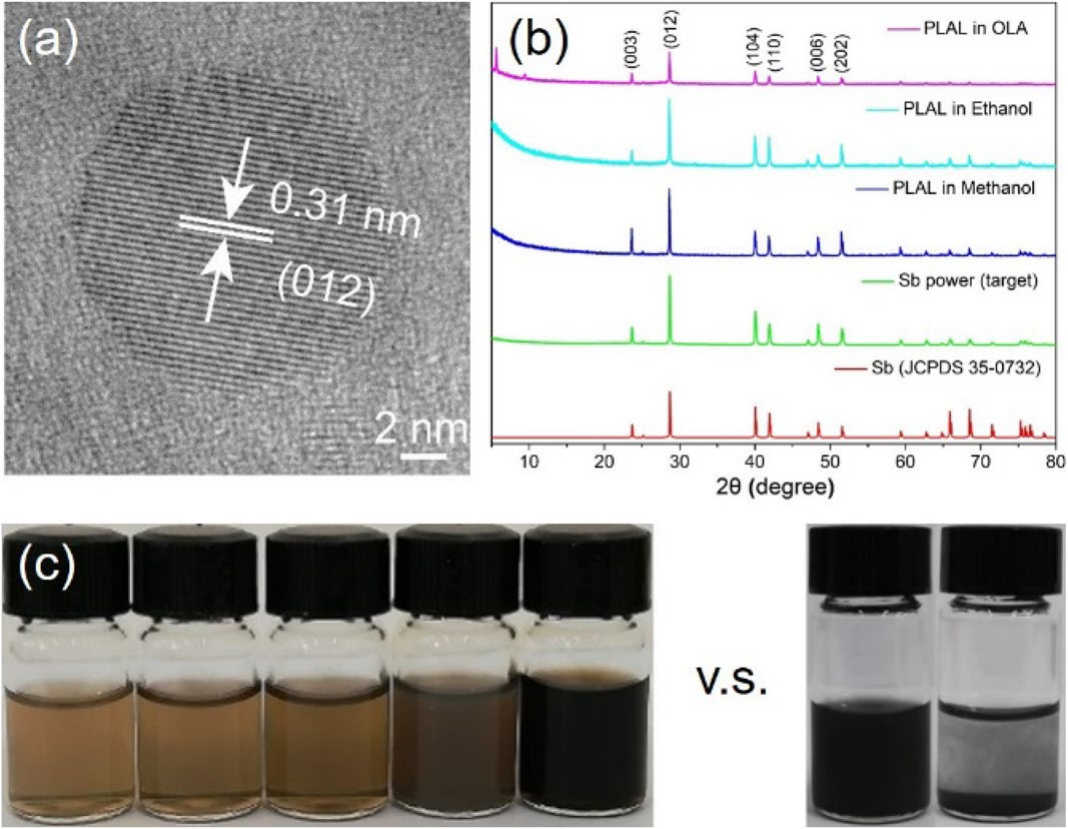
(**a**) HRTEM image of a Sb NC prepared by PALA, (**b**) XRD patterns of the Sb target, Sb NCs and standard rhombohedral Sb, (**c**) The stable PLAL prepared colloidal Sb NCs in various solvents, from left to right: ethanol, isopropanol, n-butanol, methanol and DMF, and v.s. Sb NCs prepared by chemical reduction in precipitated in 30 min (left: 0 min; right: 30 min).

Figure 2a shows a typical HRTEM image of the Sb NCs prepared by the PLAL method. The Sb NC is sphere shaped with good crystallization. The lattice fringes of the crystal plane are obviously discernible. The measured lattice parameter is 0.31 nm, consistent well with the (012) crystal plane of the rhombohedral structured Sb crystal. The single crystalline structure of the Sb NC extends to surface with a clear and sharp edge, indicating a relatively clean surface without an inorganic oxide shell. The XRD patterns (Fig. 2b) of the Sb target and the Sb NCs prepared by PLAL in different solvent also proved they have the rhombohedral structure (space group N166, R$\stackrel{-}{3}$m, a = b = 0.4307 nm, c = 1.1273 nm, JCPDS 35-0732). No other phases or impurities were detected, suggesting the high purity of the Sb NCs. The stable and evenly dispersibility of Sb NCs is very important because it is crucial for the following operations and applications, for examples, to prepare uniformly distributed composites, to make high quality films and to be used for biological purpose. Here by the PLAL method, this aim can be achieved simply by using many solvents. Various solvents including *N*,* N*-dimethyl-formamide (DMF), oleylamine, oleic acid, alcohols of methanol, ethanol, isopropanol, n-butanol and their mixtures were successfully used to prepare Sb NCs via the PLAL method. It was found that a relatively high yield in DMF was achieved compared with other solvents. The colloidal Sb NCs in all these solvents showed good dispersibility (Fig. 2c). In comparison, typical colloidal Sb NCs with size of 20 nm were synthesized by chemical reduction of SbCl_3_ in various solutions^27^. Although these synthesized Sb NCs could disperse in DMF by ultrasonication to form a uniform solution, they would precipitate in 30 min. The PLAL prepared Sb NCs are stable and well dispersed in the solvents over days under ambient conditions. An exceptional case is that when the PLAL were carried out in H_2_O, only white antimony oxide particles were obtained. If re-disperse the PLAL Sb NCs prepared in ethanol or DMF into H_2_O, the colour would gradually turn white–grey in 12 h, indicating partial oxidization. Further modification is thus needed to stabilize the Sb NCs in aqueous for long term applications.

**Figure 3:**
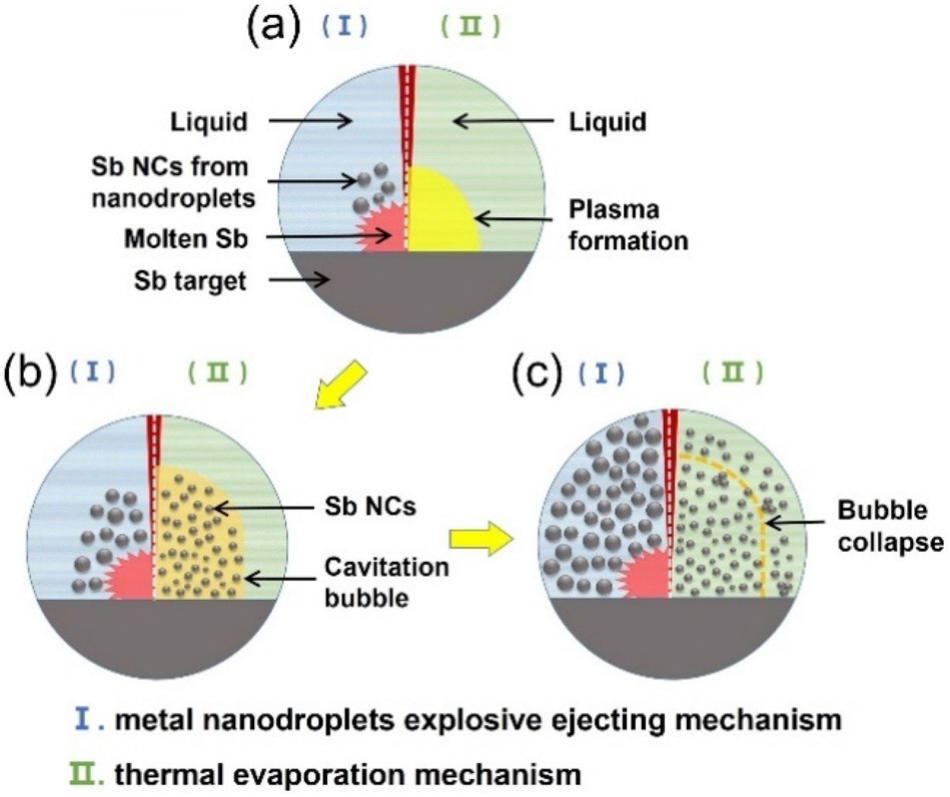
Schematic illustration of the mechanisms of the PLAL preparation of Sb NCs.

With carefully survey of the TEM images, it turned out that the size and morphology of the Sb NCs can be categorized into two types. One is the Sb NCs with the typical size of 10–50 nm, featured in nearly perfect shape of sphere and free standing from each other. The other one is the Sb NCs typically < 10 nm. These two types of Sb NCs are generally observed in all these samples. It suggests that there may exist different formation mechanisms during the PLAL process. Former studies of the PLAL physics indicate that there are two main mechanisms^43–45^. For a low power density laser of 10^6^–10^7^ W/cm^2^ with a long pulse width of millisecond, the metal nanodroplet explosive ejecting mechanism could be responsible for the nanocrystal formation, e.g. Ti, Zn, Pb NCs^46^. When the target under ablation is locally melted, the surrounding solvent boils to produce high pressure vapor which induces a strong shattering effect, resulting in the molten metal being ejected into the liquid medium at a high speed in the form of nanodroplets, which were solidified immediately by the solvent. It was reported nanosecond short pulsed laser could also produce metal nanodroplets^47,48^. Another mechanism is the thermal evaporation mechanism. The pulsed laser shot on the target produces a high-temperature and high-pressure vapor or plasma. The subsequent ultrasonic adiabatic expansion of the hot plasma led to a quick cooling of the plume region, and hence to the formation of NCs/clusters. The collapse of the plasma bubble releases the NCs/clusters to the solvent^46,49–53^. Here we attribute the bigger Sb NCs to the metal nanodroplet explosive ejecting mechanism. The nearly perfect sphere shape is consistent with the nature of the molten nanodroplets before frozen. The laser power density in this work is 7.5 × 10^6^ W/cm^2^ and pulse width is microsecond, matching with the typical explosive ejecting condition. The 0–15 nm Sb NCs are attributed to the thermal evaporation mechanism, for the plasma cooling process is more beneficial to form smaller NCs. For examples, the thermal evaporation produced Pt NCs were found smaller than the nanodroplet explosive ejection produced ones^54–59^. Although the typical thermal evaporation mechanism is commonly reported to be generated by lasers with short pulse width of femtosecond to nanosecond and high energy density (10^8^–10^10^ W/cm^2^), it is also believed to occur simultaneously with the nanodroplet explosive ejection for the existence of multimodal size distribution^46^, just as we observed for the Sb NCs. In fact, the metal nanodroplets ejection was also proposed to be driven by the expanding vapor/plasma splashing of the molten target^60^. Figure 3 shows a schematic illustration of the mechanisms of the PLAL preparation of Sb NCs.

**Figure 4:**
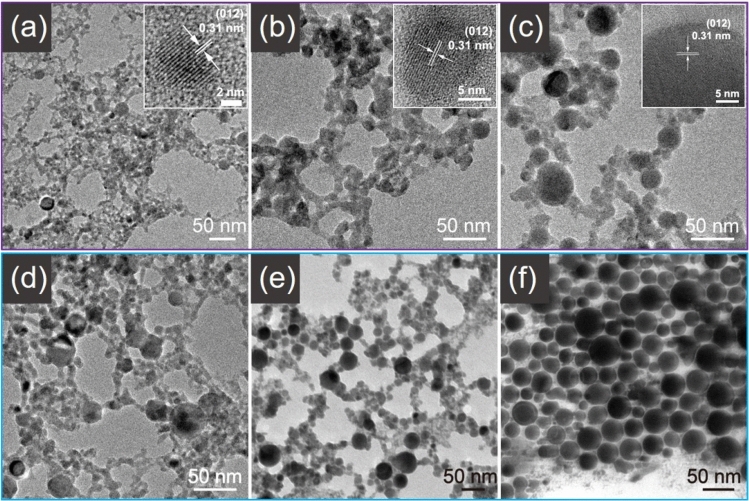
TEM images of Sb NCs prepared in ethanol with (**a**) the laser power of 30 W, (**b**) 12 W and (**c**) 6 W (inset: corresponding HRTEM images); TEM images of Sb NCs by adding (**d**) 0 g, (**e**) 0.05 g, (**f**) 2 g PVP in 10 mL DMF under 30 W.

Base on the above discussed Sb NC forming mechanisms, it can be anticipated that if the contribution ratios of the two mechanisms change, the Sb NC size (size distribution) can be tuned. TEM images (Fig. 4a–c) show that with the laser power decreased from 30 to 6 W, the dominate species changes from small (< 10 nm) Sb NCs to the bigger (10–50 nm) Sb NC spheres. It indicates that the thermal evaporation mechanism occurs at high laser power density while the metal nanodroplet ejection mechanism dominates at lower laser power density, consistent with the above mechanism discussions. Though varied in size, those Sb NCs are dispersed in solvents well, forming transparent brown colloidal solution. Realized the viscosity of the solution may affect the range of the vapor or plasma zone in the solvent, we use polyvinylpyrrolidone (PVP) as a viscosity modulator. By adding 0.05 g PVP to the DMF, the Sb NC spheres significant increased; when up to 2 g PVP added, all the product was Sb NC spheres (Fig. 4d–f). This phenomenon suggests that the addition of polymer thickening agent would prohibit the thermal evaporation process, while the nanodroplet ejection mechanism is not affected. It was found that different from the laser power, the scanning speed in 200 to 1,000 mm/s range has little effect either on the size of Sb NCs or on the product yield. In the low speed side, the next laser pulse spot is mostly overlapped with the former; In the high speed side, the next laser pulse spot apart from the former. It was reported that the next laser pulse spot apart from the cavitation bubble generated by the former but within its heat and shock wave affected zone may help enhance the yield^61^. However, it was not obvious in our experiment. It may be due to (1) the high cooling efficiency of the ablation in liquid media, given the cooling time is much longer than the ablation time (pulse width of 1 μs and frequency of 5 kHz), and/or (2) fast heating up ratio of each laser pulse compared with the laser pulse width that leading to the Sb NC formation less sensitive to the Sb target surface either in solid or molten state.

**Figure 5:**
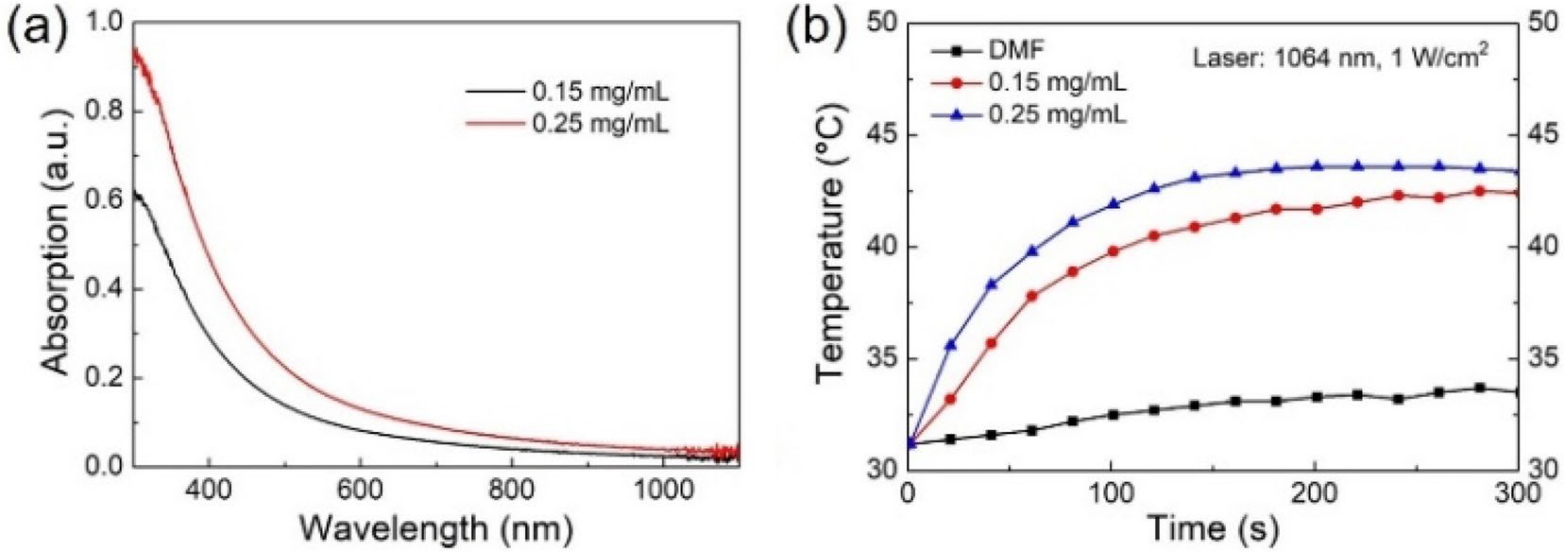
(**a**) Optical absorption spectra, (**b**) near infrared photothermal property of the PLAL Sb NCs with a concentration of 0.25 mg/mL and 0.15 mg/mL.

The UV–Vis-NIR absorption measurement shows that the absorption spectra of the PLAL Sb NCs extent to the near infrared range (Fig. 5a). Therefore, it could be a good candidate for near infrared thermal therapy. The photothermal performance of the PLAL Sb NCs was investigated (Fig. 5b). The temperature of the Sb NCs solution in DMF with a concentration of 0.25 mg/mL rapidly increased from 31.2 to 43.3 °C after irradiation at 1 W/cm^2^ for 120 s; at concentration of 0.15 mg/mL the temperature increased to 40.5 °C under the same irradiation condition. This is a relatively high photothermal performance under near infrared light of 1,064 nm, comparable to that of the carbon dot passivated black phosphorus nanosheet hybrids^62^. It should be mentioned that for most photothermal nanomaterials^63–70^, and for all the reported Sb nanocrystals or antimonene as photothermal therapy agent, the illumination wavelength of 808 nm in the NIR-I window was used^18,19,21,22^. Since the 1,064 nm is in the NIR-II region, it could have better through skin and bone performance. Therefore, it may find specific applications in cases such as through scalp and skull photothermal therapy of deep orthotopic brain tumors^71^. Zhang et. al reported a unique concept of using photothermal property of black phosphorus at hydrogels loaded with drugs for cancer therapy. The black phosphorus converts light into heat that softens and melts the hydrogel-based nanostructures, with the releasing drugs been accurately controlled by the NIR light intensity, exposure duration, black phosphorus concentration and hydrogel composition^72^. Similar strategies could also be adopted for Sb NCs.

## Conclusions

Nearly spherical colloidal Sb NCs were successfully prepared by PLAL method. Compared with chemical synthesis of Sb NCs, this is a general method that can be readily carried out in various solvents. The as prepared Sb NCs are dispersed well forming brown colored stable colloidal solutions. A combined formation mechanism of metal nanodroplet explosive ejection and thermal evaporation was demonstrated. The former results 10–50 nm Sb NCs while the later yields < 10 nm Sb NCs. The ratio of these two types of Sb NCs can be controlled by the solvent and laser ablation power. The good near infrared photothermal property in the NIR-II window suggests its promising potentials for applications in biomedical or photothermal treatment. It could be combined with other bio-functional nanomaterials^73,74^. For the therapy of persistent tumors, combinatorial strategies of photothermal-chemotherapy, photothermal-immunotherapy, photothermal-gene therapy and photothermal-radiotherapy also bring about new opportunities addressing the challenges of single-mode phototherapy^75^. Most importantly, the photothermal effect can be used not only for therapy, but also for environmental applications^76,77^, such as photothermal evaporation for clean water production, photoinduced antimicrobial treatment, photocatalytic applications.

## Supplementary information

Supplementary Legend.

Supplementary Video.
