# Supplementary material for: Continuous preparation of antimony nanocrystals with near infrared photothermal property by pulsed laser ablation in liquids
Source: Sci Rep. 2020 Sep 15;10:15095. doi: 10.1038/s41598-020-72212-2 (PMC7493941; doi:10.1038/s41598-020-72212-2)
Supplement: Supplementary file 1 — Supplementary Legend. [file 41598_2020_72212_MOESM1_ESM.docx]

Supplementary Information

Continuous Preparation of Antimony Nanocrystals with Near Infrared Photothermal Property by Pulsed Laser Ablation in Liquids

Juanrong Kou^1^, Yongkai Wang^1^, Xiaoyu Liu^1^, Xianju Zhang^1^, Gaoyu Chen^1^, Xiangxing Xu^1,*^, Jianchun Bao^1^, Kaili Yang^2^ & Lihui Yuwen^2,*^

^1^School of Chemistry and Materials Science, Nanjing Normal University, Nanjing, 210046, China.

^2^Key Laboratory for Organic Electronics and Information Displays & Jiangsu Key Laboratory for Biosensors, Institute of Advanced Materials (IAM), Jiangsu National Synergetic Innovation Center for Advanced Materials (SICAM), Nanjing University of Posts and Telecommunications, Nanjing, 210023, China.

^1,*^Email: xuxx@njnu.edu.cn, ^2,*^Email: iamlhyuwen@njupt.edu.cn

**Supplementary information**:

File name: Supplementary Video.mp4

Video content: continuous preparation of Sb NCs by Pulsed Laser Ablation in Liquids
